# Using an Automated Speech Recognition Approach to Differentiate Between Normal and Aspirating Swallowing Sounds Recorded from Digital Cervical Auscultation in Children

**DOI:** 10.1007/s00455-022-10410-y

**Published:** 2022-01-29

**Authors:** Thuy T. Frakking, Anne B. Chang, Christopher Carty, Jade Newing, Kelly A. Weir, Belinda Schwerin, Stephen So

**Affiliations:** 1 Research Development Unit, Caboolture Hospital, Metro North Hospital & Health Service, McKean St, Caboolture, QLD 4510 Australia; 2 Centre for Clinical Research, School of Medicine, The University of Queensland, Herston, QLD 4029 Australia; 3 Speech Pathology Department, Gold Coast University Hospital, Gold Coast Hospital & Health Service, 1 Hospital Boulevard, Southport, QLD 4215 Australia; 4 Department of Respiratory Medicine, Queensland Children’s Hospital, 501 Stanley St, South Brisbane, QLD 4101 Australia; 5 Child Health Division, Menzies School of Health Research, Charles Darwin University, PO Box 41096, Casuarina, NT 0811 Australia; 6 Australian Centre for Health Services Innovation, Queensland University of Technology, Level 7, 62 Graham St, South Brisbane, QLD 4101 Australia; 7 Griffith Centre of Biomedical and Rehabilitation Engineering, Menzies Health Institute Queensland, Griffith University, Gold Coast, 4222 Australia; 8 School of Engineering and Built Environment, Griffith University, Parklands Dr, Southport, QLD 4215 Australia; 9 Menzies Health Institute QLD & School of Health Sciences & Social Work, Griffith University, Gold Coast Campus, 1 Parklands Avenue, Southport, QLD 4222 Australia; 10 Allied Health Research, Gold Coast University Hospital, Gold Coast Hospital & Health Service, 1 Hospital Boulevard, Southport, QLD 4215 Australia

**Keywords:** Cervical auscultation, Deglutition, Swallowing sounds, Classifier, Machine learning, Aspiration

## Abstract

Use of machine learning to accurately detect aspirating swallowing sounds in children is an evolving field. Previously reported classifiers for the detection of aspirating swallowing sounds in children have reported sensitivities between 79 and 89%. This study aimed to investigate the accuracy of using an automatic speaker recognition approach to differentiate between normal and aspirating swallowing sounds recorded from digital cervical auscultation in children. We analysed 106 normal swallows from 23 healthy children (median 13 months; 52.1% male) and 18 aspirating swallows from 18 children (median 10.5 months; 61.1% male) who underwent concurrent videofluoroscopic swallow studies with digital cervical auscultation. All swallowing sounds were on thin fluids. A support vector machine classifier with a polynomial kernel was trained on feature vectors that comprised the mean and standard deviation of spectral subband centroids extracted from each swallowing sound in the training set. The trained support vector machine was then used to classify swallowing sounds in the test set. We found high accuracy in the differentiation of aspirating and normal swallowing sounds with 98% overall accuracy. Sensitivity for the detection of aspiration and normal swallowing sounds were 89% and 100%, respectively. There were consistent differences in time, power spectral density and spectral subband centroid features between aspirating and normal swallowing sounds in children. This study provides preliminary research evidence that aspirating and normal swallowing sounds in children can be differentiated accurately using machine learning techniques.

## Introduction

Up to one third of children with paediatric feeding disorders have identified oropharyngeal aspiration (abbreviated to aspiration), where fluids, foods and/or saliva enter the trachea below the level of the true vocal cords pre-, during and/or post-swallowing [1]. Clinical signs and symptoms of aspiration can be overt (e.g. reflexive cough generated to expel aspirated material) or considered silent when there is an absence of cough within 20 s of the aspiration event [2]. The prevalence of silent aspiration in infants and children is between 80 and 89% in children with paediatric feeding disorders [3, 4]. The accurate detection of aspiration, including silent aspiration, is important in infants and children because missed detection of aspiration can lead to acute and chronic lung sequelae [5–7], reduced nutritional growth and development [8] and decreased caregiver health-related quality of life [9].

In current clinical practice, a videofluoroscopic swallow study (VFSS) is the preferred instrumental assessment for aspiration in infants and children due to the ability to visualise all phases of the swallow, including direct visualisation of aspiration events [10]. However, VFSS is not readily available, involves exposure to ionising radiation (although at safe levels) [11–13] and may not replicate typical mealtimes due to time constraints associated with minimisation of screening time [14], observations of eating and drinking in an unfamiliar environment and difficulties replicating typical infant formula or breastmilk with barium impregnated fluids [15, 16]. Paediatric VFSS is also limited by accessibility due to the high costs associated with medical imaging equipment and the requirement of multiple health professionals specifically trained in conducting and interpreting paediatric VFSS [14]. As such, cervical auscultation has the potential to complement instrumental assessment and facilitate assessments which are more representative of typical mealtimes for infants and children.

Cervical auscultation (CA) is the most commonly used adjuvant to the clinical feeding evaluation across the United Kingdom, Ireland and Australia [17, 18]. CA is a repeatable, non-invasive technique that uses a stethoscope, digital accelerometer or digital microphone to capture swallow and breath sounds generated during the oral preparatory, oral and pharyngeal phases of swallowing [19, 20]. The use of CA has demonstrated high sensitivity 0.85 (95% CI 0.62–0.97) and negative predictor value 0.92 (95% CI 0.78–0.98), as well as good to very good reliability (inter-rater kappa = 0.81; 95% CI 0.79–0.84, intra-rater kappa range 0.72–0.98) in detecting aspirating swallows [21, 22]. Recent studies have also shown that specific sound features of swallow [23] and post-swallow breath [19] sounds can accurately differentiate between aspirating and non-aspirating swallows in children based on digital cervical auscultation.In recent years, machine learning has gained popularity in health care for diagnostics and outcome prediction. Machine learning involves using statistical algorithms to model underlying relationships between variables (or features), to make predictions. For diagnostic applications, supervised machine learning approaches are most common because the algorithms are trained to classify the presences or absence of disease or dysfunction. Support Vector Machine (SVM) algorithms have gained popularity in the machine learning community as they can provide accurate predictions when the relationship between the features and the outcome are non-linear. SVM classifiers are considered the most appropriate for use in sound classification, given its non-linear ability to differentiate binary classes (i.e. aspiration versus non-aspiration) using feature selection techniques on smaller datasets [24–26]. To date, radial basis classifiers have been shown to accurately detect aspirating swallows in children with paediatric feeding disorders related to neurological conditions (sensitivity of 79.4% [27] and 92.2% [28]). These studies [27, 28] demonstrated that using machine learning can accurately classify aspiration in children but 100% diagnostic test accuracy remains elusive.

Previous studies using machine learning are limited by several factors including their use of pre-processed sounds and/or pre-selected input features to classify aspirating swallows. Pre-processed sounds refer to the process of removing contaminating signal components which are not related to swallowing sounds. This may include signal (e.g. cough, breath sounds) or disturbance noises (e.g. vasomotion of major arteries, head movement) [29]. Using pre-selected mathematical or physiological features to classify aspirating swallow sounds in adults likely resulted in unintentional bias and reduced accuracy of the classifier of previous CA studies [30]. Also, previous classifier accuracy results were based on swallowing sounds collected from children with large age ranges (2–11.6 years [27] and 2–9.9 years [28]) and on a variety of fluid viscosities. These are limitations as age and viscosity influences swallowing kinematics and acoustic swallowing parameters of amplitude, frequency and duration for children [31–33]. To minimise confounding factors of age and viscosity on swallowing sounds, studying swallowing sounds in children of a narrow age range and on single viscosities are necessary. Additionally, symptoms of paediatric swallowing disorders can be heterogeneous and nonspecific to underlying medical aetiology; therefore, an approach which can accommodate such heterogeneity whilst detecting key attributes of aspiration swallowing sound features has the potential to increase the accuracy in detecting aspiration amongst infants and children.

Automatic speaker recognition (ASR), an approach which aims to identify a human speaker from a digital recording of their voice, may provide increased accuracies to the detection of aspirating swallow sounds in children. In conventional ASR, the speech signal is represented as temporal changes in the shape of the human vocal tract, which contains speaker-specific information that can be used to identify a person. Engineering models of the vocal tract shape mathematically represent the resonant frequencies as the spectral envelope of the power spectral density of the speech signal [34]. The frequency locations of strong spectral peaks or formants, which represent resonances in the vocal tract, convey information on the speech content as well as the speaker characteristics [35]. Based on a similar premise, aspirating swallow sounds are the result of fluid flow into the trachea, below the level of the vocal cords. It is therefore reasonable to hypothesise that formant-based speech features, such as LPCCs (or linear prediction cepstral coefficients) [36], MFCCs (or Mel-frequency warped cepstral coefficients) [37] and PLPs (or perceptual linear prediction) [38], could convey useful information for discriminating aspirating from non-aspirating swallowing sounds using machine learning techniques. To date, CA has differentiated between safe and unsafe swallows [39], swallow function [40] and correlated specific swallow kinematic events such as hyoid bone displacement [41–43] and laryngeal closure [44] in adults. Given that ASR collates signals based on the temporal changes in the shape of the human vocal tract and the promising correlates of CA to specific swallow kinematics in adults, the differentiation of aspirating from non-aspirating swallowing sounds may be possible. This study aimed to investigate the accuracy of using an ASR approach to differentiate between normal and aspirating swallowing sounds recorded from digital cervical auscultation in children.

## Methods

This study was approved by a Human Research Ethics Committee. Data analyses were completed on two groups of children where participants were prospectively recruited and informed consent obtained from the caregivers and assent from older children.

### Participants

We had two groups of children: (i) typically developing and (ii) feeding disorders.

Group (i) was recruited from the generally community, median age 13 months (range 4–33 months, 52.1% males). Their inclusion criteria were as follows: aged 4–36 months and confirmation of normal oral feeding development via a Pre-Feeding Checklist [45] for infants aged 4–7 months or confirmation of normal oromotor functioning via the Schedule for Oral Motor Assessment (SOMA) [46] for children aged 8–36 months. Children were excluded if they had a medical history of any of the following: developmental delay, visual/hearing impairment, neurological impairment, aerodigestive tract structural abnormalities, genetic syndromes, paediatric feeding disorder, neurodevelopmental disorder and/or prematurity (< 37 weeks of gestation) [31]. Children included in this study were chosen based on closest match in age (months) to Group (ii).

Children from group (ii) were aged 2–71 months (median age 10.5 months, 61.1% males). Diagnoses included congenital syndromes (e.g. Beckwith-Wiedermann, Cru de Chat, Pierre Robin Sequence), neurological (e.g. cerebral palsy), respiratory (e.g. bronchiectasis, chronic cough) anatomical anomalies (e.g. oesophageal atresia, congenital myopathy, tracheoesophageal fistula) and other (e.g. developmental delays, failure to thrive).

### Procedure

#### Normal Swallows from Typically Developing Children Group

This dataset consisted of 106 sound clips of thin fluid initial and subsequent swallows from 23 healthy infants/children. Depending on the children’s preferences and gross motor development, children were seated upright at a small chair and table, in a high chair or positioned on their caregiver’s lap. Children were fed by their caregiver, the researcher or allowed to independently feed themselves depending on their expressed preferences and/or fine motor skills development. Children were first offered three single bites or single sips of age-appropriate food/fluid consistencies in a standardised order of puree, lumpy mash, chewable solids and thin fluids. Bolus sizes were not standardised to allow children to eat and drink bolus volumes which replicated their typical mealtime experiences. After this, children were allowed to free eat and drink the remainder of food/fluids for a period of 30 min.

#### Aspirating Swallows from Children with Paediatric Feeding Disorders Group

The second dataset consisted of 18 thin fluid swallows from 18 patients with confirmed aspiration via VFSS with various underlying medical aetiologies. Depending on the children’s age and level of gross motor development, children were seated either semi-reclined or upright on a tumbleform chair, which was positioned on top of a Videofluroscopic Imaging Chair. Children were viewed in the lateral position and offered a standard VFSS protocol of two presentations of puree, lumpy mash, chewable solid, extremely thick, moderately thick, mildly thick, slightly thick and/or thin fluids by either their caregiver or a speech pathologist. The order of the protocol was consistent for all children; however, adaptations to the type of foods presented were made based on individual medical status and feeding development of each participant. Conferral on the presence/absence of aspiration was jointly completed by a paediatric speech pathologist and radiologist; and objectively rated by a speech pathologist post-VFSS, using the Penetration–Aspiration Scale [47]. Scores of 6, 7, or 8 were considered aspirating swallows due to the entry of material below the level of the vocal cords. Only one swallow on thin fluids per patient was used in this study. Aspirating swallows used were chosen based on the absence of extraneous noises as a result of a loss of flat contact of the microphone resulting in capture of ambient room noise.

### Equipment

**Fig. 1:**
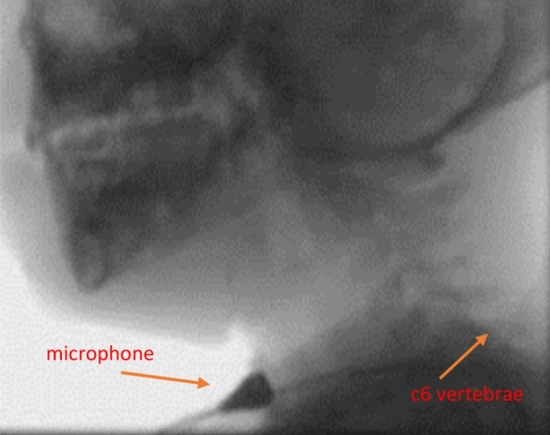
Placement of microphone during a videofluoroscopic swallow study (VFSS)

An omnidirectional condenser microphone (C417, AKG Acoustics, Vienna, Austria) (sensitivity at 1 kHz of 10 mV/Pa, impedance 200, frequency range 20 to 20,000 Hz) was placed on the skin surface lateral (< 1 cm) to the cricoid cartilage aligned at the level of the 6^th^ cervical posterior vertebrae (Fig. 1). Palpation of the cricoid cartilage was performed prior to microphone attachment to help guide accurate placement for all participants. To ensure the microphone was consistently placed, all were placed by the principal researcher only using a fitted circular O-ring and secured with microfoam tape. The use of a circular O-ring and microfoam tape maximises flat surface contact, reduces pick up of ambient room noise and allows the infants and children to move as they typically would during mealtimes without interfering on sound quality. All swallowing sounds were digitally recorded (Digital H4n Handy Recorder, Zoom Corporation, Tokyo, Japan) and were continuously monitored prior to and throughout the recordings using headphones (Model ATH-M50, Audio-Technica, Taiwan). For quality control, adjustments to the microphone placement and taping were made for the detection of extraneous noises (e.g. loss of flat contact resulting in capture of ambient room noise) and/or loss of accurate microphone placement (e.g. children pulling off the microphone).

Visual images of children drinking thin fluids were captured via a digital video-recorder (Model DCR-DVD605E, Sony Corporation, Tokyo, Japan) for the normal group, whilst VFSS images were captured at 15 frames per second via a digital fluoroscopy unit (Toshiba KXO-80G, North Ryde, NSW, Australia) for the aspiration group. Visual images for both groups were all simultaneously recorded on the Digital Swallowing Workstation (KayPentax, Pentax, New Jersey, USA). Where possible, nasal airflow direction was also simultaneously recorded via placement of an infant or paediatric sized nasal cannula, which was secured firmly behind the ears. A vocal signal was used at the beginning of all assessments to enable accurate synchronisation of acoustic and visual data. Visual and audio recordings of children drinking thin fluids were downloaded from the Digital Swallowing Workstation (KayPentax, Pentax, New Jersey, USA) onto an external hard drive and synchronised on video-editing software (Sony Vegas Movie Studio 9, Madison, WI). Manual segmentation for time points of normal swallows were completed by a research assistant and clinician researcher with speech pathology experience trained to identify the start and end points of the swallows. The start point of a swallow sound was defined as the commencement of a fluid flushing sound (audio data) and commencement of laryngeal motion associated with pharyngeal activity (visual data). The end point of a swallow was defined as the cessation of a fluid flushing sound (audio data), combined with no laryngeal motion (visual data). Inter-rater reliability for swallow segmentation was performed on a random selection of 25% of swallows and intra-class coefficients of > 0.99 were obtained for both raters [31, 48].

### Preprocessing and Feature Extraction from Swallowing Sounds

**Fig. 2:**
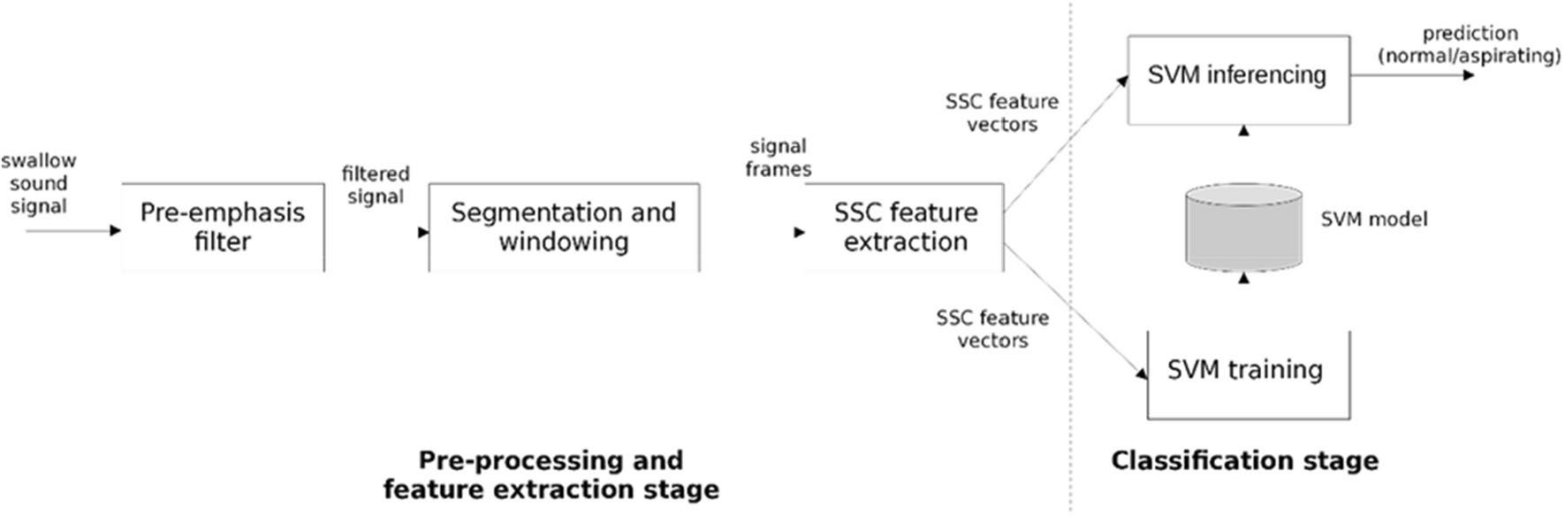
Flowchart of the pre-processing, feature extraction, and classification stages

An overview of the processing that was performed is summarised in Fig. 2. No downsampling was applied to the swallowing sounds to record the entire dynamic range. In the feature extraction stage, the swallow sounds were passed through the following pre-emphasis high-pass filter.

$$H\left(z\right)=1-0.97{z}^{-1}$$

The purpose of the pre-emphasis filter was to compensate for any spectral tilt by flattening the power spectrum so that higher frequency formants were emphasised [36]. The filtered sounds were then segmented into 20 ms overlapping frames with a 10 ms update and a Hamming window was applied to each frame.

Feature vectors were then computed for each frame. There are several speech features that are commonly used in automatic speaker recognition, such as LPCCs, MFCCs and PLPs. The purpose of the feature extraction process is to derive useful parameters of the spectral envelope shape of the power spectrum, specifically, the frequency locations of the formants [35]. Whilst MFCCs are very popular features used in conventional ASR, they are also well known to be sensitive to additive noise. Early experiments as part of this study were performed using MFCCs to identify aspirating sounds; however, results showed relatively poor classification performance. Rather than using cepstral-based features, which utilise both formant and non-formant regions of the power spectrum, it was discovered that features based on formant locations, specifically, spectral subband centroids (SSCs) [49], were better suited for this task.

To compute SSCs, the power spectrum is filtered by a Mel-frequency warped triangular filterbank (similar to that used in MFCCs), which results in *M* subbands, where *M* is the number of triangular filters used. In this study, *M* was set to 26. The spectral centroid frequency of each subband is computed and these centroid frequencies together form a *M*-dimensional SSC feature vector. Spectral subband centroids tend to be more robust because they are more heavily influenced by the location of the formants, which can be less sensitive to broadband noise [49].

Based on features from a previous study on SVM classification of acoustic events [50], a feature vector for each patient is then formed by computing the mean and standard deviation of the SSC vectors and concatenating them together, giving the final feature dimension of 52.

### SVM Classifier Design

There was a total of 124 swallows, of which 18 were aspirating and 106 normal swallows. Training and test sets were formed using a 50/50% stratified random split, which ensured the same distribution of classes in both sets. The training and test set each comprised 53 normal and 9 aspirating swallows. Since SSCs are frequency locations that are required to be in ascending order, feature standardisation was not applied. The class labels for normal and aspirating swallows were set to 0 and 1, respectively.

The classifier used in this study was the support vector machine (or SVM) [51], which is well suited to situations where training data are limited. The SVM is a large margin classifier that has good generalisation properties and is relatively robust to outliers, since the computed hyperplane that separates the two classes is determined only by a smaller subset of training vectors known as the support vectors. By using different SVM kernel functions, which effectively transform the feature vectors into a higher dimensional vector space in order to achieve better class separation, a non-linear decision hyperplane can be efficiently computed [52].

The SVM classifier in the Python library scikit-learn [53] was utilised in the experiments for training and testing. In order to determine the optimal SVM parameters, such as the type of kernel (sigmoid, radial basis function or polynomial), kernel parameters (gamma and polynomial order) and regularisation parameter *C*, a grid search was performed using a five-fold cross-validation on the training set. In this study, the best parameters found were *C* = 1 using a second order polynomial kernel. To handle the class imbalance problem, the regularisation parameter *C* for each class was weighted by a factor that was inversely proportional to the class frequencies, as is implemented in the “balanced mode” of the SVC in scikit-learn.

## Results

**Fig. 3:**
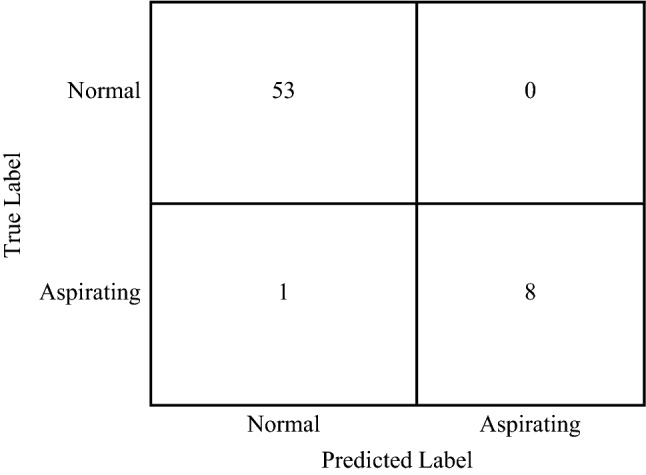
Confusion matrix between normal and aspirating classes

Confusion matrices summarises prediction results on a classification problem and often used in the field of machine learning. The confusion matrix of the normal (0) and aspirating (1) classes for the SVM classifier are shown in Fig. 3. This matrix shows that of the true normal swallows, the classifier correctly identified 53 as normal swallows, and 0 as aspirating. Of the true aspirating swallows (*n* = 9), the classifier labelled 1 as a normal swallow and 8 as aspirating swallows. The matrix demonstrates that the proposed method accurately identified all normal swallows with only one false negative, i.e. the classifier identified an aspirating swallow as normal. Table 1 lists the overall performance of the SVM classifier, where the total accuracy was found to be 98%.

**Table 1:** SVM classifier performance between patients with aspirating and normal swallows (total accuracy = 0.98)

	PPV or precision	NPV	Specificity	Sensitivity or recall	F_1_ score
Normal swallow (0)	0.98	1.00	0.89	1.00	0.99
Aspirating swallow (1)	1.00	0.98	1.00	0.89	0.94

The F_1_ score, which is defined as the harmonic mean of the precision and recall, is another measure of accuracy that is more conservative, balances the contribution of false negatives and false positives to the final metric, and is better suited to cases where classes are unbalanced

*PPV* positive predictive value, *NPV* negative predictive value

**Fig. 4:**
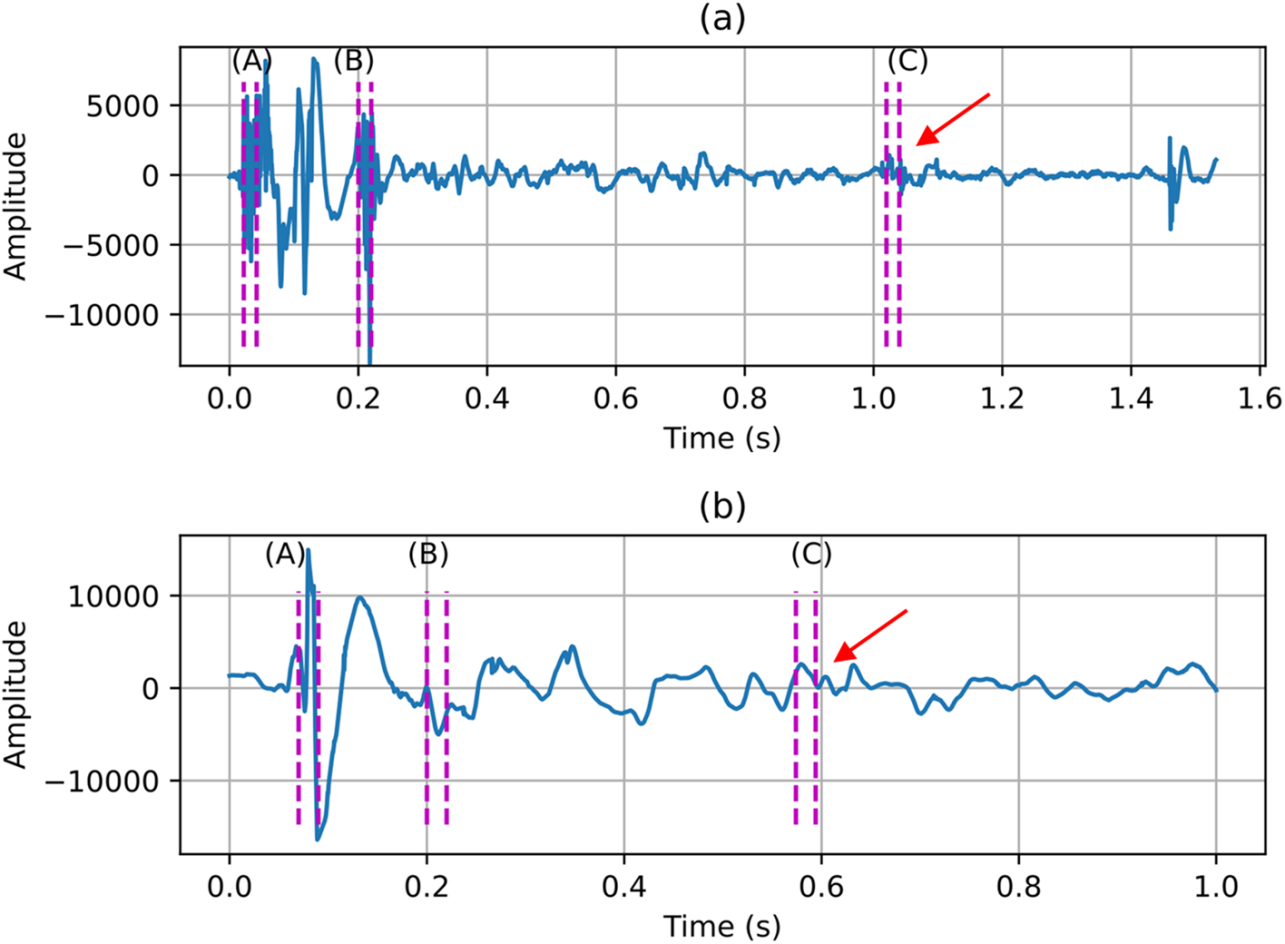
Time domain representations of and regions of interest (A, B, C) in **a** an aspirating swallow; and **b** a normal swallow

**Fig. 5:**
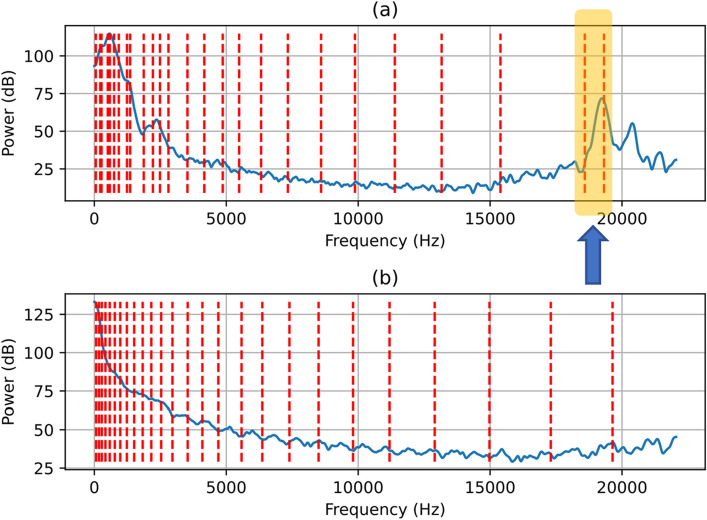
Power spectral density after pre-emphasis (blue line) and SSC frequencies (dashed red lines) of region (A) from Fig. 4: **a** an aspirating swallow; and **b** a normal swallow

**Fig. 6:**
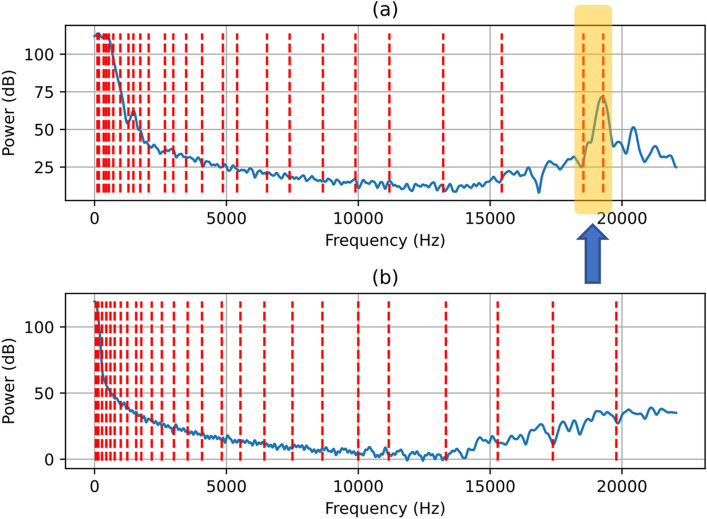
Power spectral density after pre-emphasis (blue line) and SSC frequencies (dashed red lines) of region (B) from Fig. 4 in: **a** an aspirating swallow and **b** a normal swallow

**Fig. 7:**
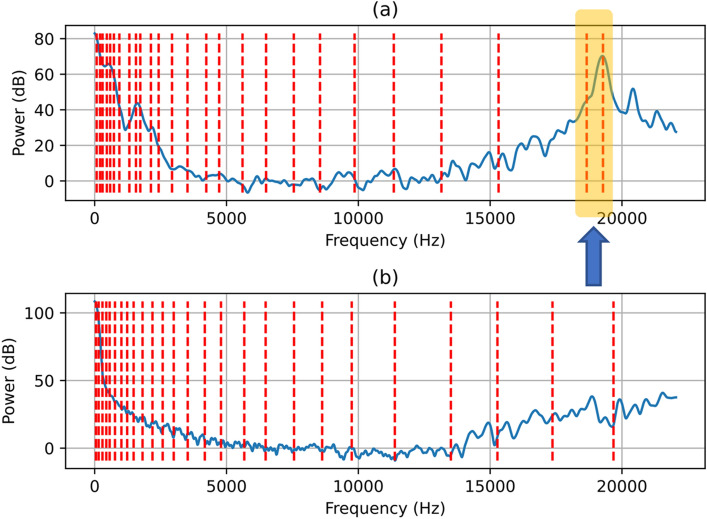
Power spectral density after pre-emphasis (blue line) and SSC frequencies (dashed red lines) of region (C) from Fig. 4 in: **a** an aspirating swallow and **b** a normal swallow

Aspirating swallow sounds had different time domains and power spectral density (PSD) features compared to normal swallows. To demonstrate this difference, Fig. 4 shows randomly selected time domain segments (each with labels for three points of interest A,B and C) of aspirating swallows (subplot 2(a)) and normal swallows (subplot 2(b)). The corresponding PSD for both aspirating and normal segments and each point of interest A, B and C are shown in Figs. 5, 6 and 7. In Figs. 5, 6 and 7, the dashed red vertical lines indicate the location of the individual SSCs. Since the SSCs are computed as the centroid of different frequency subbands, they will tend to be more concentrated around strong spectral peaks. This higher concentration of SSCs can be seen in the low frequencies where the spectral components are strong. Therefore, the SSCs, which are highlighted in Figs. 5, 6 and 7, are able to capture the high-frequency spectral peak that is near the 20 kHz, which appears to be a characteristic of the power spectral density of the aspirating swallow sound.

**Fig. 8:**
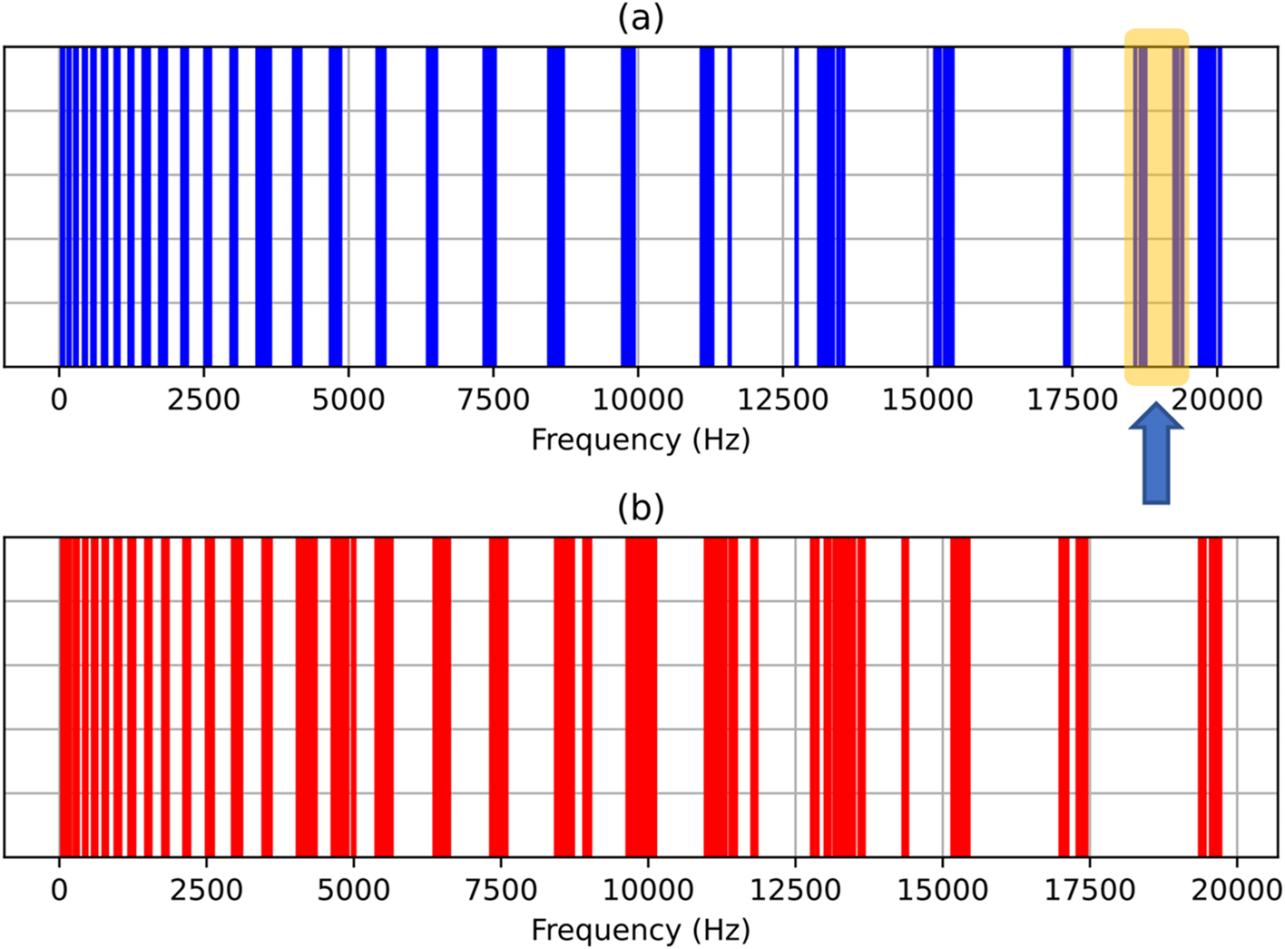
Plot of mean SSCs for all **a** aspirating swallows (18) and **b** normal swallows (106). There are 26 “bands” that represent the 26 SSC features. Within each band, a vertical line represents the mean SSC for an individual swallow sound. The highlighted region in **a** shows SSCs that only appear in aspirating swallow sounds

In order to determine whether these characteristic SSCs are a common feature that can be used for the discrimination between normal and aspirating swallow sounds, Fig. 8a and b shows all the SSCs from the entire aspirating and normal swallow sounds dataset. As shown in the highlighted region in Fig. 8a, there are high-frequency SSCs that only appear in aspirating swallow sounds.

## Discussion

To our knowledge, this is the first study to use an ASR approach to determine a classifier for the differentiation between normal swallows in healthy children and aspirating swallows in a cohort of children with paediatric feeding disorders using digital CA. High accuracy for the detection of aspirating and normal swallows were found, with sensitivities of 89% and 100%, and NPVs of 1.00 and 0.98, respectively. We demonstrated differences in time, power spectral density and SSC features between aspirating and normal swallows in children.

Previous studies investigating the use of a classifier to detect aspirating swallow sounds in children have documented lower overall accuracies of 79.8% [27] and 89.6% [28] when compared to the overall accuracy of 98% found in our study. It is possible that the lower accuracies previously reported were at increased risk of type I errors due to multiple swallow sound data points collected and used from each participant with a paediatric feeding disorder. The same studies also used swallow sounds recorded via an accelerometer on a combined range of fluid viscosities and solid consistencies [27, 28]. Exclusively focussing on thin fluids is essential in the development of a swallowing sounds classifier because there are known differences in the acoustic properties (e.g. intensity, duration, frequency) between thin and puree bolus’ in children [32] and adults [54, 55]. Thus, our higher reported accuracy may have been facilitated by exclusively using thin fluid swallowing sounds across two independent groups of healthy children and children with paediatric feeding disorders, thereby minimising type I error and the heterogeneity of swallowing sounds.

Our reported sensitivity of 89% for the detection of aspiration is higher than previously documented sensitivities of between 33 and 92% for routinely performed clinical feeding evaluations in children when compared with gold standard tests, such as the VFSS or fiberoptic endoscopic evaluation (FEES) [10, 56–63]. In comparison with cervical auscultation-specific diagnostic test accuracy data in children, our overall sensitivity for the differentiation between aspirating and normal swallows is superior to subjective clinician judgement when cervical auscultation was used in conjunction with the clinical feeding evaluation 85% (95% CI 0.62–0.97) [21] or in isolation 93.9% (95% CI 91.8–95.6) [22]. The improvements in accuracy for the differentiation between aspirating and normal swallows found in our study are likely attributed to the ASR approach used. Given that the ASR approach is based on temporal changes to the shape of the vocal tract, it is plausible that the entry of fluids into the trachea and below the level of the vocal cords caused changes to features of the swallowing sound which would not be audible on normal swallows.

In our study, consistent differences in time, power spectral density and SSC features were found between aspirating and normal swallows in children. For aspirating swallow sounds, the power spectral density at specific swallowing time points contained strong peaks in the high frequencies when compared to normal swallowing sounds. In addition, only a specific SSC feature appears in aspirating swallowing sounds and is absent from all normal swallowing sounds. We hypothesise that the SSC and power spectral density features on aspirating swallow sounds are one out of 21 physiologic components in the oropharyngeal mechanics of feeding/swallowing in infants [64, 65]. Martin-Harris and colleagues [65] completed the largest known prospective study on 300 infants which investigated the quantification of swallowing function. Based on the gold standard VFSS and validity testing, Martin-Harris and colleagues [65] demonstrated that aspiration formed one of five domains of swallowing in infants. As such, the contributions of our preliminary work of SSC and power density features lay the foundation for future approaches to develop an aspiration classifier for swallowing sounds which correlate with quantifiable physiological components of the paediatric feeding/swallowing mechanism.

Whilst our study uniquely described the use of machine learning to accurately classify aspirating swallow sounds in children, there are limitations to our study. Firstly, VFSS was not used to objectively classify swallowing sounds obtained in the normal group of children. Our team acknowledge that obtaining ethical approval and caregiver consent for radiation exposure with VFSS for healthy infants and children are unlikely. Instead, we used the results of the SOMA [46] and/or pre-feeding checklists [45] to objectively categorise normal feeding/swallowing skills. Secondly, our small sample of aspirating swallows (*n* = 18) was a limitation and there is the potential for overfitting based on this small specific dataset. To overcome any bias towards overfitting, we used stratification and class weightings in the SVM to preserve the class balance between aspirating and normal swallows. This ensured consistent proportions of the two swallow types used for machine learning. Future studies with larger sample sizes of aspirating swallows in children with paediatric feeding disorders are required to further validate use of the ASR approach for the classification of aspiration. In addition, comparative studies of different classifiers are required to provide reliable interpretation of reported diagnostic accuracies which are performed on the same dataset to reduce known bias’ in machine learning within healthcare [66].

The potential benefits of an accurate classifier for the detection of aspiration based on swallowing sounds provide an important step towards cervical auscultation being a complementary diagnostic instrumental assessment to VFSS. Cervical auscultation has the potential capability of helping health professionals worldwide working in the field of paediatric feeding disorders to personalise feeding management plans which are patient and family centred [67] due to its non-invasive nature, low cost and repeatability in multiple environments. However, further studies are required which uses machine learning principles to systematically understand swallowing sound properties in a variety of clinical populations in infants and children. Speech pathologists continue to have an important role in the assessment of aspiration risk and the complex richness of information (e.g. breath sounds, respiratory status, positioning) that is obtained in a clinical feeding evaluation whilst further work on refining current swallowing classifiers continues.

## Conclusion

This study provides preliminary evidence that the use of machine learning techniques could accurately classify aspirating swallowing sounds collected from digital cervical auscultation in children with a high degree of accuracy (98%), sensitivity (89%) and PPV (100%).
